# Prevalence of Proximal Contact Loss between Implant-Supported Prostheses and Adjacent Natural Teeth: An Umbrella Review

**DOI:** 10.1055/s-0042-1745771

**Published:** 2022-06-10

**Authors:** Amirhossein Fathi, Ramin Mosharraf, Behnaz Ebadian, Mehdi Javan, Sabire Isler, Sara Nasrollahi Dezaki

**Affiliations:** 1 Department of Prosthodontics, Dental Materials Research Center, School of Dentistry, Isfahan University of Medical Sciences, Isfahan, Iran; 2 Department of Prosthodontics, Dental Implants Research Center, School of Dentistry, Isfahan University of Medical Sciences, Isfahan, Iran; 3 Private Practice, Tehran, Iran; 4 Department of Prosthodontics, Istanbul University, Istanbul, Turkey; 5 Dental Students' Research Committee, School of Dentistry, Isfahan University of Medical Sciences, Isfahan, Iran

**Keywords:** proximal contact loss, implant-supported prostheses, adjacent natural teeth

## Abstract

Contact loss between the implant prosthesis and adjacent natural teeth is a clinical complication whose overall prevalence is uncertain. Therefore, the main purpose of this umbrella study was to evaluate the extent of contact loss between implant prostheses and adjacent natural teeth. Electronic database of MEDLINE/PubMed, Cochrane, and Google Scholar was searched until August 2021 without considering language restrictions and according to Preferred Report Items for Systematic and Meta-Analysis guidelines (preferential reporting items for systematic review and meta-analysis). Inclusion criteria were systematic/meta-analysis review articles related to contact loss between implant prostheses and adjacent natural teeth. Inclusion criteria and risk of bias for the selected systematic/meta-analysis review studies were assessed by two or three qualified researchers, and the fourth researcher was used to resolve the ambiguities. From 43 eligible articles, five systematic/meta-analysis review studies were selected for this study. Important information such as the range of contact points, the prevalence, and the location of the contact loss was extracted. Three research studies had a low risk of bias and were considered clinical evidence. Analysis of low-risk studies showed that the superiority of open contact loss was excessive. Prevalence of proximal contact loss was more in mesial contact, especially in the mandibular arch. No significant differences were reported in sex or between the posterior and anterior regions.

## Introduction

The use of implant-supported prostheses in patients suffering from edentulousness is a good treatment with a good prognosis. 1 2 Nevertheless, various biological and mechanical complications are reported in implant prostheses 3 4 5 6 7 ; one of the most important complications is interproximal contact loss (ICL) between the implant prosthesis and the adjacent natural tooth, 8 9 and numerous physiological factors play a role in the occurrence of this complication, including the type, location, and chewing forces. In fact, the most important issue regarding this complication is the mesial migration of natural teeth, 10 11 for which periodontal ligament is responsible.

Osseointegrated implant prostheses are ankylosed and unmovable, which result in further contact loss. 12 13 However, there is no consensus on the underlying cause of ICL since it is not seen in all patients necessarily. 14 It is difficult to avoid ICL due to its various possible factors which may be progressive and require clinical intervention. To prevent the displacement of food particles, caries, and periodontal disease, modification of the prosthesis and restoration of adjacent natural teeth are suggested as ICL 15 16 17 treatment methods. Some studies have mentioned that ICL causes inflammation around the implant, resulting in marginal bone loss and implant failure. 14 18

Considering the importance of the issue, this umbrella review investigates the incidence of ICL between implant prostheses and nearby natural teeth. In this study, the null hypotheses were that “there is no correlation between implant-supported prostheses and natural teeth adjacent to the ICL” and “there is no significant difference between the sex of the individual and the ICL position (mesial/distal, anterior/posterior, and maxilla/mandible).”

## Methods

### Search Strategy

Electronic searches were conducted in PubMed/MEDLINE, Cochrane, and Google Scholar until August 2021 without language restrictions. According to population, intervention, comparison, and outcome), the research question was “Is there a correlation between contact loss of the implant prosthesis and the nearby natural tooth?.” The “population” included patients with implant prostheses. The “intervention” consisted of contact loss between implant prostheses and adjacent natural teeth. “Comparison” was performed with individuals who had contact with the implant prosthesis and adjacent natural teeth. The primary “outcome” included the prevalence of ICL between implant-supported prostheses and adjacent natural teeth; the secondary “outcome” included the incidence of ICL according to gender and positions. This review study was conducted using the Preferred Report Items for Systematic and Meta-Analysis guidelines. 19 The articles used included a systematic/meta-analysis review and resources that examined the incidence of contact loss among implants and adjacent natural teeth. The Assessment of Multiple Systematic Reviews (AMSR2) method was used to calculate the risk of systematic/meta-analysis review bias. 20 The database was searched based on medical subject heading (mesh) and non-mesh keywords in simple or multiple conjunctions: ((((dental implant [Title/Abstract]) OR (implant with support [Title/Abstract])) AND (loss of contact [Title/Abstract])) OR (open contact [Title/Abstract])) OR (adjacent natural teeth [Title/Abstract]).

### Inclusion and Exclusion Criteria in Screening

Inclusion criteria consisted of clinical studies in systematic/meta-analysis review and the existence of contact loss between implant-prostheses and nearby natural teeth. Exclusion criteria included duplicate reviews, comments, and editorials. The studies were confirmed following receipt of the full text and observation of the inclusion and exclusion criteria.

### Data Collection Process

Two independent reviewers (R.M. and B.E.) qualified the eligible articles for review (1.0 Kappa). One researcher (R.M.) was responsible for extracting qualitative or quantitative data from the studies, and the second researcher (B.E.) was responsible for reviewing all collected data. Collected information included the author's name, year and type of the study, the number of contacts, the incidence of contact loss, location, and type of prosthesis. Ambiguity and incompatibility were solved by resolving discussions. If a problem were unresolved, the third researcher helped. The initial search yielded 43 articles, of which 17 remained after removing duplicates and irrelevant ones by consensus. Five studies were found eligible eventually.

### Bias Risk Assessment

Based on the risk of bias assessment, to assess the quality of systematic/meta-analysis review studies, we used 16 questions of AMSR2 20 ( Table 1 ). In the end, each article received a score that indicated the risk of bias in that study. With eight to eleven positive responses, the risk of bias decreased; if four to seven questions were answered positively, the risk of bias was moderate and if fewer than three questions received a positive response, the risk of bias was considered as high. 21 Three qualified investigators assessed the articles (kappa = 0.9). Ambiguity and incompatibility were followed by resolving discussions. If a problem remained unresolved, the fourth researcher assisted.

**Table 1:** The Assessment of Multiple Systematic Reviews (AMSR2) tool

Systematic articles		1	1	3	5	6	7	8	9	11	14	2	4	10	12	13	15	16
		2	1	3	5	6	8	11	14	4	7	2	9	10	12	13	15	16
		3	1	2	3	5	6	10	11	13	14	16	4	7	8	9	12	15
		4	1	2	3	5	6	9	11	12	13	14	16	4	7	8	10	15
		5	3	16	1	2	4	5	6	7	8	9	10	14	11	12	13	15
	AMSR2 items																	
		Yes					Partial yes					No					NMC	

Abbreviation: NMC, no meta-analysis conducted

Note: Overall methodological quality: low: 0–5, moderate: 5–10, and high: 11–16.

Note: Criteria for AMSTAR analysis according to positives answers: low risk (8–11), moderate risk (4–7), and high risk (≤3).

## Results

### Screening of Systematic/Meta-analysis Reviews

**Fig. 1:**
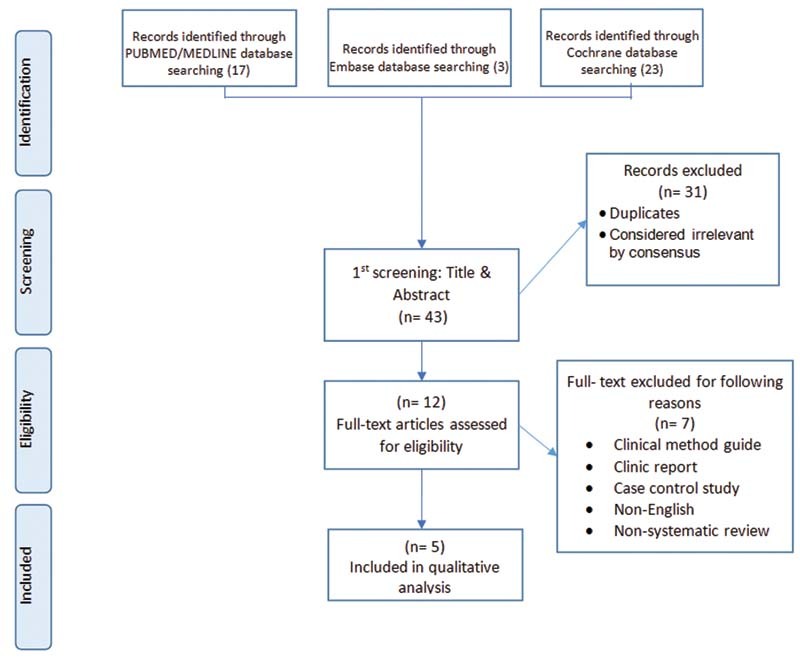
Flow charts for the studies were identified, displayed, and included in the study.

A search in PubMed/MEDLINE databases (17 articles), Embase (three articles), Google Scholar (zero), and the Cochrane Library (23 articles) resulted in finding 43 articles. After removing duplicate sources, 41 studies remained for reviewing the titles and abstracts. After carefully studying the titles/abstracts of the articles, 12 articles met the eligibility criteria. Seven of them 15 16 17 22 23 24 25 were excluded due to the reasons indicated in Table 2 , and a total of five studies were selected eventually, 14 26 27 28 29 which included 73 articles published between 2021 and 2016. Details of the research strategy are in Fig. 1 . Table 3 summarizes the most important features of these studies.

**Table 2:** Excluded studies and reasons for exclusion

	**References**	**Reasons for exclusion**
22	Kim et al 2019	Clinical method guide
16	Liu et al 2019	Clinic report
17	Sfondouris and Prestipino 2019	Clinic report
23	Zeng et al 2018	Case control study
24	Luo et al 2016	Non-English
25	Ren et al 2016	Non-systematic review
15	Wat et al 2017	Clinic report

**Table 3:** Baseline characteristics of systematic reviews assessing the prevalence of proximal contact loss between implant-supported prostheses and adjacent natural teeth

Author (y)	Types/No. of **s** tudies **i** ncluded	Number of contact loss	Method of **a** nalysis	Search **p** eriod	Population	Interventions	Comparison	Outcomes assessed	Risk of **b** ias	Main results
Bento et al 2021 26	8 retrospective Two prospective	6,473	Systematic review and meta-analysis	Up to September 2020	Patients rehabilitated with implant-supported prostheses	The proximal contact loss between the implant-supported prostheses and adjacent natural teeth	Situations in which no proximal contact was lost between the implant-supported prostheses and adjacent natural teeth.	The prevalence of proximal contact loss between implant prostheses and adjacent natural teeth, the prevalence of proximal contact loss in terms of sex and location	Low risk	The prevalence of proximal contact loss was high, occurring more frequently with the mesial contact and the mandibular arch. Significant differences were not found concerning sex or between the posterior and anterior region
Manicone et al 2021 27	10 retrospective Five prospective	12,370	Systematic review and meta-analysis	November 2020	Patients who had received or were scheduled to receive a single implant restoration had been rehabilitated or were scheduled to be rehabilitated with implant-supported fixed partial dentures	The overall prevalence of PCL determines the distribution and clinical features	Prevalence, the condition, context, and population framework.	The number of cases of PCL that occurred, the number of PCL that occurred at the mesial contact point, at the distal contact point, in the mandible, and the maxilla	Moderate risk	PCL is a frequent complication. Approximately 29% of contact points develop this condition, which may cause food impaction and damage to the interproximal tissues
Oh et al. 2020 28	11 retrospective Five prospective	2,757	Systematic review and meta-analysis	March 2020	Patients with implant-supported prostheses or tooth-supported prostheses	Open proximal contact with implant-supported fixed prostheses compared with tooth-supported fixed postheses	Odds of developing OPC with implant-supported prostheses or tooth-supported prostheses	Odds of OPC between implant-supported fixed prostheses or adjacent teeth	Low risk	The odds of developing OPC were significantly higher with implant-supported prostheses than with tooth-supported prostheses
Papageorgio et al 2018 29	Nine RCTs 14 retrospective Four prospective	7,664	Systematic review and meta-analysis	June 2018	Human patients of any age, sex, or ethnicity with at least one osseointegrated dental implant placed (including its restoration) among natural teeth	Frequency of infra-position and missing contact points in implant-supported restorations within natural dentitions over time	The influence of various patient, implant, or study-related characteristics	The IIP of the osseointegrated implant (and its supra-structure) compared with adjacent teeth, to loss of the PCP of the implant crown with the adjacent natural tooth	Low risk	Long-term adverse effects of dental implants among natural teeth can be observed in terms of IIP and PCP loss to the adjacent teeth
Greenstein et al 2016 14	Five RCTs	501	Review	2015	Patients with implant-supported prostheses or tooth-supported prostheses	Evaluate the potential causes, clinical significance, and treatment of open contacts between dental implant restorations and adjacent natural teeth	Percentage of restored dental implants manifesting open proximal contact areas adjacent to natural teeth	The incidence of open contacts that develop after implant restorations occurs next to teeth	High risk	The occurrence of an interproximal separation next to an implant restoration was greater than anticipated

Abbreviations: IIP, implant infra-position; OPC, open proximal contact; PCL, proximal contact loss; PCP, proximal contact point; RCT, randomized control trial.

### Risk of Bias Assessment

The risk of bias was measured using the AMSR2 tool. This tool uses for a variety of different studies. Based on the number of correct responses, the level of bias in the study was reported as high, medium, or low ( Table 1 ). In this study, the risk of bias was either moderate (including one systematic/meta-analysis review 27 ) or low (including three systematic/meta-analysis reviews 26 28 29 ). Systematic/meta-analysis review studies with a low risk of bias were considered as clinical evidence. The low-risk systematic/meta-analysis review accounted for 72.6% of the study volume ( Table 3 ).

### Characteristics of Systematic Reviews

General information on each systematic/meta-analysis review is presented in Table 3 (authors and year of publication, number, and type of studies, type of analysis, research period, interventions, outcomes, risk of bias, and main results).

### General Sample Analysis

Generally, we analyzed five review studies that consisted of 73 articles (43 retrospective, 16 prospective, and 14 RTC articles) and 29,765 (501 to 12370) proximal contact points in total. All reviews 14 26 27 28 29 reported a high prevalence of ICL; Oh et al 28 showed that the ratio of ICL in the implants was 2.5 times higher than that of the teeth and was increased in proximal space in follow-up periods; Manicone et al 27 showed that ICL is a common problem occurring in 29% of contact points associated with an increase in inflammation of adjacent teeth. One of the reviews 26 declared that the posterior/anterior regions and gender did not affect the prevalence. However, most reviews 26 28 29 stated high heterogeneity and the need to perform further randomized control trials and blinded observations.

Further examination of the studies showed that the incidence of ICL in the maxillary areas was similar to that of the mandible. 30 31 32 33 34 35 36 37 Besides, all studies presented more damage in the mesial regions. Some articles 30 32 33 34 36 compared ICL in the anterior and posterior regions and concluded that ICL mostly occurs in the posterior regions. 32 33 34 Most studies evaluated age, sex, and implant site and did not find any significant correlation between appearance of ICL and these factors.

## Discussion

This umbrella study examined the incidence of ICL between implant prostheses and nearby natural teeth. The null hypotheses were not accepted because the results showed more ICL between implant-supported prostheses and nearby natural teeth. There was also a significant difference in the position of the ICL (mesial/distal).

The main result of this umbrella review was a high incidence of ICL among natural teeth and implant prostheses. Studies have also concluded that the prevalence of ICL increases more than 80% after 5 years. 38

ICL is a common complication. According to Manicone et al, it occurs in approximately 30% of contact points. Open contact (OC) is annoying to the patient, causes more inflammation in nearby tissues, and can increase the risk of new defects. 27 The prevalence of proximal OC varied between studies. 9 15 33 36 37 Various studies have reported the first OC between 1 and 123 months after the restoration. 33 36 37 Wong et al indicated that the incidence of OC was similar among prostheses repaired with screw or cement. As time goes on, the size of the space between the teeth and the implant restoration may increase 15 33 and the number of OC will increase over time. 9 33 36 37

Studies have reported that the prevalence of ICL among different sexes and ages is almost the same, indicating that ICL is not limited to a specific age or gender. 39 These studies indicate that 50 µm metal shim and flossing can affect the prevalence of ICL. Possible causes of ICL include dental migration, artificial crown-related factors, and bone growth factors. 31 33 34 37

The results of this umbrella study show that the mesial ICL is more prevalent than the distal. The possible cause of this condition is faster wear in the mesial than distal. Thus, the mesial naturally compensate for this wear through displacement. The clinical causes of ICL in the distal region are not well understood. In general, the results of studies indicate that osseointegrated implants are at risk of infra-occlusion and mesial/distal ICL obstruction due to the destruction of nearby teeth and bone growth in the facial region. 40

Some studies have reported a higher prevalence of ICL in the mandible than in the maxilla. 14 37 This may be due to the tendency of the mandibular teeth to mesial and, thus, increase the likelihood of developing ICL. 33 Studies have reported a similar incidence of ICL in the anterior/posterior regions. In patients with a high Frankfort-mandibular plane angle, the anterior force components are high and increase the prevalence of ICL. According to the anterior force theory, when a force applies to the posterior teeth, this force travels through the proximal contacts to reach the midline. Therefore, this force transmission causes wear in the proximal contact of the anterior and posterior teeth.

ICL can be a high-risk complication in implant-supported restorations because it causes marginal bone resorption. 30 32 The results suggest that patients should be aware of the increased risk of ICL among natural teeth and implant-supported prostheses over time. 32 34 The causes of ICL are not fully understood, so more research is needed. One of the limitations of this umbrella study is the high heterogeneity between articles, such as the type of study, non-randomness, different methods, and durations of ICL evaluation which makes it difficult to determine the prevalence and evaluate the adverse effects of ICL.

## Conclusion

Based on the findings of this umbrella study, the following general results are included.

ICL is very common and often occurs at the contact points in mesial and in the mandibular arch, and no significant differences were reported between ICL and gender or posterior/anterior regions.
